# Creating a new mental health e-cohort using the SHARE research register: Linking questionnaire, genetic and routine health data.

**DOI:** 10.23889/ijpds.v7i3.2054

**Published:** 2022-08-25

**Authors:** Matthew Iveson, Mark Adams, Andrew McIntosh

**Affiliations:** 1 The University of Edinburgh

## Objectives

Advancements in mental health research depend upon continuing development of the data landscape, including the creation of population cohorts that combine different types of data from a variety of sources. The present project aimed to produce a new, multi-faceted mental health e-cohort to benefit further research.

## Approach

Taking advantage of a standing research resource – the SHARE Scotland research register (N ~ 285,000) – we administered an online survey of mental health, treatment use and wellbeing. Over 10,000 individuals (Mean age = 57.16 years; 63% female) took part in the survey; all participants consented to secure linkage of their routinely-collected health records with questionnaire data and over 90% also consented to the research use of genetic data gained from diverted blood samples. We linked questionnaire responses with routinely-collected health data and genetic data within a Trusted Research Environment to create a large cohort enhanced for mental health research.

## Results

In this presentation we describe the cohort, summarise responses to the mental health questionnaire and give some example research uses of the linked data. Focussing on depression, we contrast the prevalence of self-reported diagnoses of depression (27% of the sample) with diagnostic codes from routinely-collected hospital admission and national prescribing data and examine predictors of each.

## Conclusion

Estimates of multimorbidity prevalence vary markedly depending on how many LTCs are counted, with absolute differences in estimated prevalence largest for people in middle-age and with lowest SES. Further research is needed to support implementation of a uniform approach to defining how many conditions to include in multimorbidity measures.

